# Prevalence of five tick-borne bacterial genera in adult *Ixodes scapularis* removed from white-tailed deer in western Tennessee

**DOI:** 10.1186/s13071-014-0473-y

**Published:** 2014-10-22

**Authors:** Sarah E Mays, Brian M Hendricks, David J Paulsen, Allan E Houston, Rebecca T Trout Fryxell

**Affiliations:** Department of Entomology and Plant Pathology, University of Tennessee, 370 Plant Biotechnology Building 2505 E J Chapman Drive, Knoxville, TN USA; Department of Forestry, Fisheries, and Wildlife, University of Tennessee, Tennessee and Ames Plantation, Grand Junction, Knoxville, Tennessee USA

**Keywords:** *Ixodes scapularis*, Tennessee, Tick-borne disease, *Anaplasma*, *Babesia*, *Borrelia*, *Ehrlichia*, *Rickettsia*

## Abstract

**Background:**

In the northeastern and midwestern regions of the United States *Ixodes scapularis* Say transmits the causal agents of anaplasmosis (*Anaplasma phagocytophilum*), babesiosis (*Babesia microti*), and borreliosis (*Borrelia burgdorferi* and *B. miyamotoi*). In the southeastern United States, none of those pathogens are considered endemic and two other tick-borne diseases (TBDs) (ehrlicihosis and rickettiosis) are more common. Our objective was to determine baseline presence and absence data for three non-endemic bacterial agents (*Anaplasma, Borrelia* and *Babesia*) and two commonly reported bacterial agents (*Ehrlichia,* and *Rickettsia*) in southern *I. scapularis* (n = 47) collected from 15 hunter-harvested white-tailed deer (*Odocoileus virginianus*) in western Tennessee*.*

**Findings:**

Of the 47 ticks, 27 tested PCR positive for non-pathogenic *Rickettsia* species, two for *Ehrlichia ewingii,* one for *Ehrlichia* sp. “Panola Mountain”*,* and one for *Anaplasma phagocytophilum* variant 1 strain. None of these ticks were positive for *Babesia* or *Borrelia* (including *B. burgdorferi*).

**Conclusions:**

Finding human pathogens in host-fed *I. scapularis* merits additional studies surveying pathogen prevalence in questing ticks. Collection of questing *I. scapularis* in their peak activity months should be undertaken to determine the overall encounter rates and relative risk of pathogenic *Ehrlichia* in southern *I. scapularis. Ehrlichia* sequences were homologous to previous human isolates, but neither *Babesia* nor *B. burgdorferi* were identified in these ticks. With the identification of pathogenic bacteria in this relatively small collection of *I. scapularis* from western Tennessee, the study of the absence of Lyme disease in the south should be refocused to evaluate the role of pathogenic *Ehrlichia* in southern *I. scapularis*.

## Findings

*Ixodes scapularis* Say (blacklegged tick) is known to transmit the causal agents of many diseases causing illness and death in humans, livestock, pets, and wildlife [1]. These diseases include anaplasmosis, babesiosis and Lyme borreliosis [2–5]. Ehrlichiosis is another notable tick-borne disease (TBD), and although the causal agents *Ehrlichia chaffeensis* and *E. ewingii* have not been reported in *I. scapularis* in the U.S., an *Ehrlichia muris*-like species has been isolated from *I. scapularis* [6], and pathogenic *Ehrlichia* species have been found in other members of the *I. ricinus* complex, such as *I. pacificus* in California [7,8], *I. ricinus* in Russia [9], and *I. persulcatus* in Korea [10]. *Ixodes scapularis* has also previously been shown to be infected with spotted fever group *Rickettsia* (SFGR) species of undetermined pathogenicity [11].

In Tennessee, Lyme disease is considered non-endemic; consequently, it is not likely to pose a high risk for human infection [12,13]. Other TBDs have a greater prevalence in Tennessee, including ehrlichiosis and rickettsiosis, with Rocky Mountain spotted fever (RMSF) being the most highly-reported TBD in the state [14–16] and TBD diagnoses has been increasing steadily in Tennessee for approximately the last 5 years [16]. While *I. scapularis* frequently bites humans in northeastern and midwestern states, it is less common in southeastern states and rarely encountered on humans during summer months [17]. There is still a critical need to characterize the potential causal agents in *I. scapularis* and corroborate findings that *B. burgdorferi* is rare in the southeast; consequently, we attempt to think “beyond Lyme” as described in Stromdahl and Hickling [17] within southeastern collected *I. scapularis*. The objective of this study was to determine baseline presence and absence data for three non-endemic bacterial agents (*Anaplasma*, *Borrelia* and *Babesia*) and two commonly reported bacterial agents (*Ehrlichia,* and *Rickettsia*) in *I. scapularis* collected from white-tailed deer.

In the fall of 2011 and 2012, 17 ticks (9 engorged) were collected from 6 deer, and 30 ticks (14 engorged) were collected from 9 deer, respectively, for a total of 47 adult *I. scapularis* (21 males and 26 females) from 15 white-tailed deer harvested at Ames Plantation Research and Education Center located in southwestern Tennessee. In the laboratory, ticks were identified to life stage, species, and sex [18]. Each specimen was bisected longitudinally with a sterile scalpel blade. Half of each tick was placed in 80% ethanol and stored at -20°C as a voucher specimen. From the remaining half of each tick, total genomic DNA was extracted using a Fermentas DNA extraction kit (Thermo Scientific, Pittsburg, PA). Extracted DNA was stored at -20°C in elution buffer until screening. Extracted DNA was screened with 16S primers [19] to verify that extraction was successful and samples that could not be morphologically identified were 16S sequenced (these samples were confirmed as 99% homologous to *I. scapularis* GenBank L43855).

All 47 samples were screened for infection with *Anaplasma, Babesia, Borrelia, Ehrlichia* and *Rickettsia* species using genus-specific PCR reactions with positive and negative (sterile water) controls. *Anaplasma* and *Ehrlichia* species were amplified using *groEL* primers in a nested PCR [20] with a positive control of *E. chaffeensis* amplified from extracted tick DNA (94% homologous to GenBank L10917). Primers for *16S* rRNA were used to identify *Anaplasma* strains [21]. For *Babesia*, the genus specific primers for *NSS* were qPCR-amplified [22] with a positive control of *B. canis* amplified from extracted dog DNA. Due to the controversy and discrepancy with *Borrelia* amplification and identification [23,24], we amplified *23S* using a real-time PCR [25] and *flaB* using a nested PCR (280 F and 754R outer reaction primers, and 301 F and 737R nested reaction primers) [26]. Positive controls were a B31-strain of *B. burgdorferi* for the *23S* real-time PCR reaction, and *B. burgdorferi* for the *flaB* nested PCR. For *Rickettsia* identification, PCR was used to amplify the *ompA* gene [27] with a positive control of an uncultured *Rickettsia* sp. amplified from extracted tick DNA (99% homologous to GenBank HM446484). General and nested PCR reactions consisted of a 50 μl reaction of 5 μl extracted DNA (2 μl of initial reaction for nested reaction), 25 μl of Maxima Hot Start Green PCR Master Mix (Thermo Scientific, Waltham, MA), and 18 μl of nuclease free water. The initial *Borrelia flaB* PCR consisted of a 27 μl reaction of 2 μl extracted DNA (1 μl of initial reaction for nested reaction), 12 μl of Hot Start Master Mix, and 11 μl of nuclease free water. PCR products were run on a 1.5% agarose gel stained with ethidium bromide. For qPCR of *Borrelia 23S*, a 20 μl reaction of 2 μl extracted DNA, 10 μl Taq Polymerase (Applied Biosystems, Grand Island, NY), 0.4 μl ROX (Applied Biosystems, Grand Island, NY) and 6.6 μl of nuclease free water was used. DNA extractions, PCR amplification, and gel electrophoresis were carried out in different locations with dedicated equipment and reagents to prevent contamination.

To remove excess primers and nucleotides from positive samples, positive amplicons were cleaned with ExoSAP-IT (Affymatrix, Inc., Cleveland, OH). The University of Tennessee Molecular Biology Resource Facility then bi-directionally sequenced the cleaned products. Sequence results were initially cleaned using Sequencher (Gene Codes Corporation, Ann Arbor, MI) and aligned with ClustalW in BioEdit (Ibis Biosciences, Carlsbad, CA). Sequences published in GenBank were used for genetic comparisons and to determine species identity. Phylogenetic trees based on Bayesian analyses were created using Bayesian Evolutionary Analysis Sampling Trees (BEAST) 1.7.5 and Fig Tree software [28] to display the associations between the amplified sequences and GenBank-published sequences.

Of the 47 *I. scapularis* collected in western Tennessee, only 20 were not PCR positive for one of the five different bacterial genera tested (Table 1). None of the ticks tested were PCR positive for *Babesia* species and none of these ticks were positive for *Borrelia* using the *23 s* qPCR. Initially, *B. burgdorferi* sensu lato PCR with *flaB* primers yielded 13 positive results. These 13 amplicons were sequenced to confirm positivity, but sequencing yielded broken fragments and unamplified sequence products. We then repeated the assay for those 13 samples and found the results to be inconsistent (i.e., negatives when once positive, or inconsistent band sizes and numbers), and thus, failed to confirm the initial suspect-positives. In both *flaB* reactions, the banding patterns of unknown tick samples were faint and sequencing attempts on those bands yielded small fragments (e.g., sequence results appeared as “NNNN…”). The positive controls in this assay produced strong bands that were successfully sequenced, indicating that the reaction worked. Comparing the concentration of *Borrelia-*nested *flaB* amplicons to other similar assays, the concentration sent for sequencing with *flaB* ranged from 0 to 12 ng/μL, while *Ehrlichia* nested concentration results ranged from 45 to 70 ng/μL. The low concentration of amplified *Borrelia flaB* (range: 0 to 12 ng/μL), the faint sample bands and strong control bands, the negative 23S qPCR, and the low annealing temperatures for the *flaB* assay (52°C) (which may permit unspecific binding) indicate those 13 samples were likely negative. Given the low sample size we may not have screened enough ticks to detect *Borrelia* in western Tennessee, rather we would need to screen ~400 specimens from a population of 1000 *I. scapularis* to find a positive sample if 1% are considered positive at a 99% confidence level. Previously, 883 *I. scapularis* from hunter-harvested deer from throughout Tennessee were screened for *Borrelia* species. None of the ticks were positive for *B. burgdorferi* and only one was positive for *B. miyamotoi* [13], now considered a human pathogen [29,30]. There is much controversy surrounding the status of *B. burgdorferi* in the southern U.S. Recently the potential role of *A. americanum* in Lyme-like illnesses in the south intensified due to a report identifying DNA sequence evidence of *Borrelia* species infection in humans and ticks in the southern U.S. [26]. This study attempted to detect *B. burgdorferi s.l.* using the same assays [26], but in the confirmed *Borrelia* vector *I. scapularis*, the results of which indicted no *Borrelia* in southeastern *I. scapularis* ticks.

**Table 1:** **PCR results of adult** ***Ixodes scapularis*** **(n =47) collected from 15 white-tailed deer harvested at AMES** ^**1**^

***Ixodes scapularis***	**No. screened (Male/Female)**	**Bacterial genera screened**					
		***Anaplasma*** **spp. (% Pos.)**	***Babesia*** **spp. (% Pos.)**	***Borrelia*** **spp. (% Pos.)**	***Ehrlichia*** **spp. (% Pos.)**	***Rickettsia*** **spp. (% Pos.)**	**Total positive(% Pos.)**
*Ixodes scapularis* collected from white-tailed deer in 2011							
Engorged	0/9	0/1	0/0	0/0	0/0	0/8	0/9
Crawling	8/0	0/0	0/0	0/0	0/0	2/0	2/0
Total	17	1 (5.9%)	0 (0%)	0 (0%)	0 (0%)	10 (58.8%)	11 (64.7%)
*Ixodes scapularis* collected from white-tailed deer in 2012							
Engorged	0/14	0/0	0/0	0/0	0/3	0/14	0/17
Crawling	12/4	0/0	0/0	0/0	0/0	1/2	1/2
Total	30	0 (0%)	0 (0%)	0 (0%)	3 (10.0%)	17 (56.7%)	20 (66.7%)
Total *Ixodes scapularis* collected from AMES white-tailed deer							
Engorged	0/23	0/1	0/0	0/0	0/3	0/22	0/26
Crawling	20/4	0/0	0/0	0/0	0/0	3/2	3/2
Total	47	1 (2.1%)	0 (0%)	0 (0%)	3 (6.4%)	27 (57.4%)	31 (66.0%)

^1^Ticks were either classified as engorged (attached with mouthparts in the host animal’s skin through physical evidence such as tissue attached to mouthparts and/or expanded idiosoma) or crawling (attached on the host, but no physical evidence of feeding). Primers targeted *groEL* for *Anaplasma* and *Ehrlichia* spp, *NSS* for *Babesia* spp, *23S* and *flaB* for *Borrelia* spp, and *ompA* for *Rickettsia* spp.

The total number positive in this table shows the number of PCR-positive results (including co-infections); consequently, the total number of infections exceeds the total number of positive ticks.

**Figure 1:**
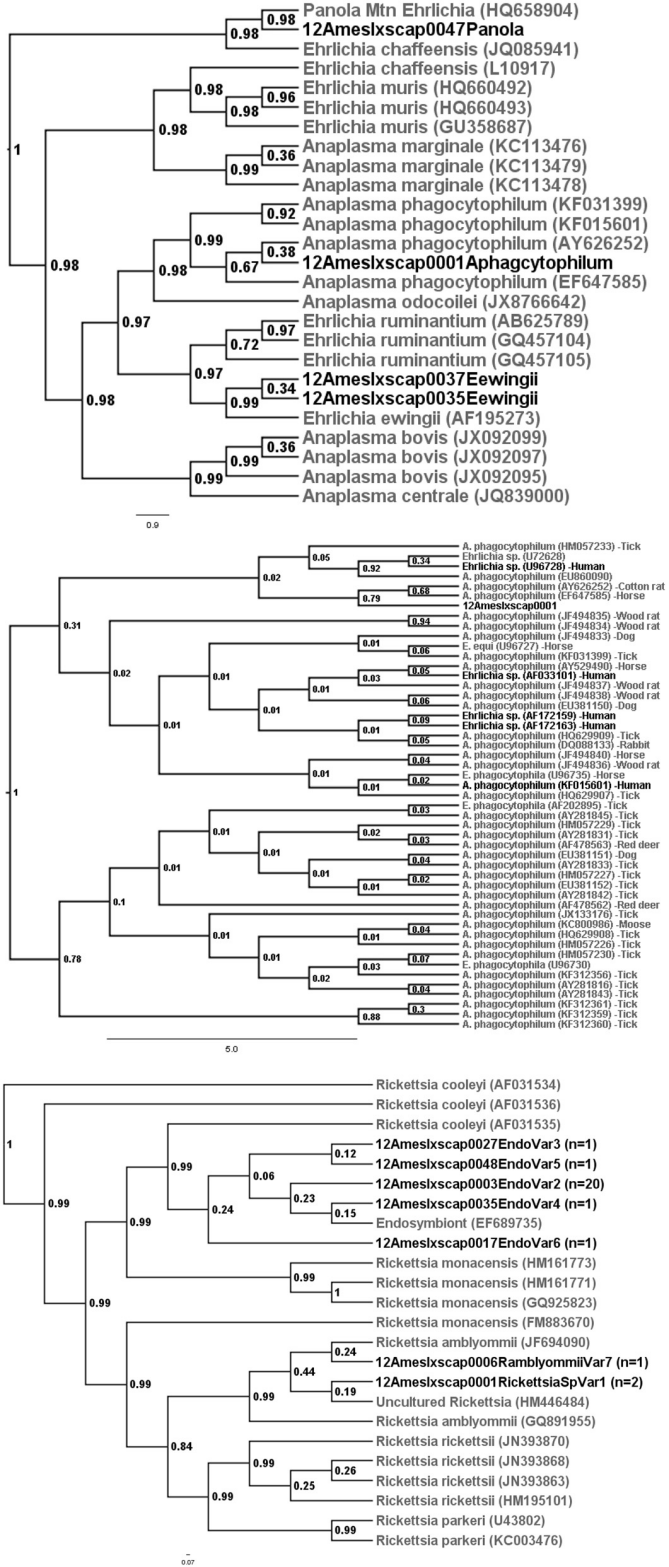
**Bayesian phylogenetic relationships of 335 bp of** ***groEL*** **amplified from** ***Ehrlichia*** **(top), 300 bp of** ***groEL*** **amplified from** ***Anaplasma*** **(middle), and 378 bp of** ***ompA*** **amplified from** ***Rickettsia*** **species (bottom) identified in** ***Ixodes scapularis*** **collected at AMES compared to reference sequences from GenBank.**

A total of 27 ticks (57.4%) were PCR positive for three different non-pathogenic *Rickettsia* species (Figure 1). Twenty-four were greater than 98% homologous to an *I. scapularis* endosymbiont (GenBank EF689735), two were greater than 98% homologous to an uncultured *Rickettsia* species (GenBank HM446484), and one was 99% homologous to *R. amblyommii* (GenBank JF694090). Due to its branch location in relation to *R. amblyommii* samples, it is likely that both the reference sequence for an uncultured *Rickettsia* spp. (GenBank HM446484) and the samples homologous to it are *R. amblyommii,* especially since these sequences were identical to the *R. amblyommii* reference sequence (GenBank JF694090) over a 380 bp region.

Although the pathogenicity of many SFGR remains undetermined, the high rates of PCR positive samples with apparently non-pathogenic SFGR has been speculated to interfere with vertical transmission of pathogenic *Rickettsia* species (such as *R. rickettsii*) and potentially reduce the risk of human rickettsiosis [4,31–34], especially since this appears to be the case for *D. variabilis* [35].

Four ticks (8.5%) produced amplicons for *groEL*, which were homologous to either *Anaplasma* species, or to *Ehrlichia* species that have been implicated as causal agents of human disease. All four of these ticks were females and at least partially engorged, suggesting the ticks acquired the bacteria from their white-tailed deer host (Table 1). One tick from 2011 had a *groEL* amplicon 99% homologous to *A. phagocytophilum* previously amplified from humans (GenBank U96728, AF033101, AF172159, AF172163 and KF015601) (Figure 1); however, subsequent testing with the *16S* rRNA primers identified it as the AP-variant 1 strain (100% homologous to GenBank AJ242784), which is not considered likely to infect humans [36]. Two ticks from 2012 were 100% homologous to *E. ewingii* previously amplified from a human patient (GenBank AF195273). One tick from 2012 was 100% homologous to *Ehrlichia* sp. “Panola Mountain” (PME) previously amplified from *A. americanum* (GenBank HQ658904) (Figure 1). Of interest to the Tennessee region, this is the first report of PME and *E. ewingii* PCR-amplified from an *I. scapularis*. All of the four ticks PCR positive for an *Anaplasma* or *Ehrlichia* species were also PCR positive (from here on defined as co-infected) with a *Rickettsia* species (Table 2). This was likely due to the endosymbiont being present in half of the ticks collected, and is not significantly greater than expected (*P* = 0.1256).

**Table 2:** **All four of the adult** ***Ixodes scapularis*** **PCR positive for** ***Ehrlichia/Anaplasma*** **(** ***groEL*** **) were also PCR positive with a** ***Rickettsia*** **species (** ***ompA*** **)**

***Rickettsia*** **and** ***Ehrlichia*** **species co-infections**				
***ompA*** **positive for** ***Rickettsia*** **species (GenBank #)**	***groEL*** **positive for** ***Ehrlichia*** **species (GenBank #)**			***Ehrlichia*** **(-) and** ***Rickettsia*** **+**
	***Anaplasma phagocytophilum*** **(# EF647585)**	***Ehrlichia ewingii*** **(# AF195273 )**	**Panola Mtn. (# HQ658904 )**	
*Rickettsia amblyommii* (#JF694090)	0	0	0	1
*Rickettsia* spp. (#HM446484)	1	0	0	1
Endosymbiont (#EF689735)	0	2	1	21
*Ehrlichia* (+) and *Rickettsia* (-)	0	0	0	0

It is important to note that detection of *E. ewingii* and PME does not incriminate *I. scapularis* in transmission of either pathogen. Since all four *Ehrlichia* and *Anaplasma*-positive ticks were feeding on white-tailed deer, (Table 1) known amplifying reservoirs of these bacteria [37–39], adults collected from deer are likely positive from the blood meal of a potentially infected host. Host blood or tissue samples were not available for confirmation of infection. These ticks collected from hunter-killed white-tailed deer are not likely to transmit either of these pathogens since they already quested for their third host and are not very likely to encounter a human host in the field. Transmission of these bacteria to humans would most likely occur after the female tick acquires the pathogen from her host, but transovarial transmission does not occur with these bacteria [34]. Observations made during collections after deer were harvested noted some of these ticks leaving the hunter-killed host and actively questing towards the collectors, indicating these ticks are capable of contacting someone processing or handling the hunter-killed deer (i.e., hunters field-dressing the animal, taxidermists, personnel at check stations). Whether *I. scapularis* can transmit one or more of these pathogens in this scenario, leaving the killed host and moving towards the hunter, remains to be understood.

From the 15 harvested deer, a range of 1 to 13 *I. scapularis* were collected and PCR screened for bacteria (Table 3). Eight deer had only one tick, and three of those eight ticks were negative for all bacteria. Three deer, identified as 2, 137, and 1210, had ticks that were PCR positive for both a *Rickettsia* and a second bacteria (*Anaplasma* or *Ehrlichia*). One deer, deer 137, had five ticks removed from it and of those five ticks, two were PCR positive for *E. ewingii,* two were positive with a *Rickettsia* endosymbiont, and one was negative for all bacteria. This was also seen in deer 1210, where two ticks were collected and both were positive for a *Rickettsia* endosymbiont and only one was positive with PME. Finding *Ehrlichia* negative ticks and *Ehrlichia* positive ticks from the same host implies that those *Ehrlichia* positive ticks may not have acquired the pathogen from that specific deer, although all ticks testing positive for an *Anaplasma* or an *Ehrlichia* species were at least partially engorged.

**Table 3:** **Infection status of adult** ***Ixodes scapularis*** **(n = 47) removed from individual white-tailed deer as determined by deer harvest number (id)** ^**1**^

**Deer Id**	**No. of Ticks**			**Infection status of ticks by individual deer**					
	**Coll.**	**Neg. (%)**	**Pos. (%)**	***Rickettsia amblyommii***	***Rickettsia*** **sp.**	***Rickettsia*** **endosymbiont**	***Anaplasma phagocytophilum***	***Ehrlichia ewingii***	***Ehrlichia*** **Panola Mountain**
*Ixodes scapularis* collected from 2011 white-tailed deer									
70	12	6 (50%)	6 (50%)	1	1	4	-	-	-
**2**	1	0 (0%)	1 (100%)	-	1	-	1	-	-
4	1	1 (100%)	0 (0%)	-	-	-	-	-	-
5	1	0 (0%)	1 (100%)	-	-	1	-	-	-
17	1	0 (0%)	1 (100%)	-	-	1	-	-	-
39	1	0 (0%)	1 (100%)	-	-	1	-	-	-
*Ixodes scapularis* collected from 2012 white-tailed deer									
123	13	7 (53.9%)	6 (46.1%)	-	-	6	-	-	-
**137**	5	1 (20%)	4 (80%)	-	-	4	-	2	-
156	3	2 (66.7%)	1 (33.3%)	-	-	1	-	-	-
96	2	1 (50%)	1 (50%)	-	-	1	-	-	-
110	2	0 (0%)	2 (100%)	-	-	2	-	-	-
**1210**	2	0 (0%)	2 (100%)	-	-	2	-	-	1
122	1	0 (0%)	1 (100%)	-	-	1	-	-	-
94	1	1 (100%)	0 (0%)	-	-	-	-	-	-
90	1	1 (100%)	0 (0%)	-	-	-	-	-	-
**TOTAL**	**47**	**20 (42.6%)**	**27 (57.4%)**	**1**	**2**	**24**	**1**	**2**	**1**

^1^Bolded deer identification numbers (identified as 2, 137, 1210) had ticks that were PCR positive with more than one bacteria. Total numbers of infections exceed the number of positive ticks due to co-infected individuals.

Finding etiological agents that are pathogenic to humans (PME and *E. ewingii*) in *I. scapularis* merits additional studies surveying pathogen prevalence of other non-*Borrelia* bacteria in questing *I. scapularis* at AMES, both because of the risk of human exposure to disease agents, and to aid in accurate diagnosis of TBD cases in western Tennessee. The potential for blood-borne transmission of *A. phagocytophilum* is also notable as it is possible that hunters may come into contact with the agent during the processing of infected deer [40], though it has been suggested that the most common strain of *A. phagocytophilum* circulating in white-tailed deer may not be pathogenic to humans [36,41]. In this study, the *Anaplasma* sequence amplified from the tick was homologous to the AP-variant 1 strain, which is not currently considered a pathogen of humans.

*Ixodes scapularis* collections in the summer months are typically infrequent in the southeastern United States [17]. In the summers of 2012 and 2013, extensive collections using dragging and dry ice trapping collected over 17,000 ticks at AMES. This number included only one *Ixodes* nymph in 2012 and three adult females in 2013. Spring and winter collections (concurrent with deer and turkey hunting seasons) have not been undertaken during the peak collection time for adults of this species, so the human risk of encountering questing adult *I. scapularis* at AMES remains unknown, but may increase in a scenario where a hunter is processing a harvested deer. Collection of questing *I. scapularis* in their peak activity months should be undertaken to determine the overall encounter rates and relative risk rates in the southeast, especially since *I. scapularis* is more widespread than previously realized [13]; however, previous studies have not indicated a large active-questing population such as those populations observed in northern areas [17,42]. Because a delay in diagnosis can be fatal, continuing education of physicians in the diagnosis of TBD is critical especially in southwestern Tennessee, where 26% of all fatal RMSF cases occur [17,43,44]. Knowledge of the disease agents present in an area and the likelihood of human exposure can be helpful in making proper diagnoses and treatment decisions, and initiating appropriate measures to prevent human infection.

## Abbreviations

AMES: Ames Plantation Research and Education Center
PME: Panola Mountain Ehrlichia
RMSF: Rocky Mountain Spotted fever
SFGR: Spotted fever group Rickettsia
TBD: Tick-borne diseases
